# Allergic Rhinitis and Its Epidemiological Distribution in Syria: A High Prevalence and Additional Risks in War Time

**DOI:** 10.1155/2020/7212037

**Published:** 2020-05-23

**Authors:** Ameer Kakaje, Mohammad Marwan Alhalabi, Ayham Alyousbashi, Aya Hamid, Osama Hosam Aldeen

**Affiliations:** Faculty of medicine, Damascus University, Damascus, Syria

## Abstract

**Background:**

Allergic rhinitis (AR) is a global disease that affects a huge proportion of people around the world especially in the Middle East, where multiple allergy-promoting factors can be found. Although AR is not fatal, it severely affects the quality of life. However, it is usually overlooked in developing countries due to resource scarcity.

**Methods:**

An online questionnaire on social media was used which included demographics, smoking, socioeconomic-status (SES), war-related questions, and the score for allergic rhinitis (SFAR), a simple self-reporting tool with the cut-off point at 7. *Findings*. This study included 968 subjects with 721 (74.5%) females. The mean age was 24.69 years with AR prevalence at 47.9%. AR was associated with male gender [*P* = 0.001 (OR, 1.677; 95% CI 1.249-2.253)], having a job [*P* = 0.049 (OR, 1.309; 95% CI 1.001-1.713)], the having a chronic medical condition (*P* < 0.0001) mainly other allergies [*P* < 0.0001 (OR, 9.199; 95% CI 3.836-22.063)] and asthma [*P* = 0.006 (OR, 5.060; 95% CI 1.396-18.342)], using medications (*P* < 0.0001) and living in particular provinces (*P* = 0.010). However, no significant correlation was found with type of work and war factors except being distressed by war sounds [*P* = 0.027 (OR, 1.348; 95% CI 1.034-1.757)]. Finally, no associations were found with age, consanguinity, SES, educational level, and cigarette or/and shisha smoking (*P* > 0.05). *Interpretation*. Approximately half of the sample displayed AR symptoms, indicating a potentially high burden of AR in the community. A correlation to being distressed from war noises was found with AR which could reflect a psychological aspect. In addition, in war harmful allergens are released which can be an additional AR risk factor which adds to the environment in the Middle East that is associated with AR. However, we need further studies to discover and minimize this huge prevalence of AR.

## 1. Introduction

Rhinitis is a respiratory disorder in the upper respiratory airway, characterized by rhinorrhoea, itching, sneezing, and nasal obstruction. Allergic rhinitis (AR) and nonallergic rhinitis (NAR) are the classifications that were previously used according to the clinical manifestations and allergic sensitization to common allergens [1]. AR occurs when nasal mucosa becomes inflamed due to allergens. Its prevalence varies from 5% to 40% of the human population worldwide [2]. AR is known to have chronic effects on more than one function of the body. Although AR is not fatal, it affects the quality of life of patients and disrupts their daily life, both socially and financially [3–5]. Risk factors may vary from smoking and drinking, domestic pet adoption, education environments, and family history to demographic factors [6–10]. Many studies suggest that the Middle East has many allergens and a higher prevalence of allergic diseases that may contribute to the high prevalence of AR [11–13]. AR can have a huge burden on the health system; in 2001 in the United States (US), AR prompted 12 million physician office visits, and the direct medical costs of AR were estimated to be around 4.5 billion US dollars per year and approximately 3.8 million lost work and school days [14]. The high prevalence of AR and its effect on many aspects of life intensify the importance of thoroughly studying AR and its risk factors, particularly in developing countries where it often goes underdiagnosed and is treated with over the counter (OTC) medications. Hence, this study aimed to assess the prevalence of AR among the developing Syrian population which has been poorly documented this far.

## 2. Materials and Methods

### 2.1. Study Design

We conducted an online cross-sectional study in Syria from 26/03/2019 to 22/04/2019. Online Arabic surveys were used that included subjects who lived in Syrian provinces. We posted the questionnaires online twice each day at 10 AM and 10 PM in several groups that covered different topics such as educational, cuisine, merchandise, entertainment, cultural, and musical. Any participant who lived in Syria, agreed on participating in the study, answered the basic demographic questions, and AR questionnaire was enrolled in this study. No further criteria was applied as it is an epidemiological study.

### 2.2. Questionnaires

- Socioeconomic status (SES): SES was assessed through three questions: the education of the person or the working family member, monthly family income, and the profession. As a result, SES was divided into 5 different categories: lower, upper-lower, lower-middle, upper-middle, and upper.
- Allergic rhinitis: We used an Arabic version of the score for allergic rhinitis (SFAR), a simple self-reporting tool with a cut-off point of 7 [2, 15]. SFAR has a sensitivity of 74% and specificity of 83% [2].
- War-related questions: We asked several questions, both directly and indirectly, about the war including changing place of residence due to war, losing someone close, and being distressed by war noises.
- Smoking: We only assessed the current smoking status for cigarettes and shisha as getting more details would be beyond this study goal. We only asked two questions in this regards, “do you regularly smoke cigarettes” and “do you regularly smoke shisha”. We did not assess for individuals who quitted smoking and the amount and time of smoking.
- Other questions: We asked basic demographic questions including gender, age, educational level, province of residency, and having consanguineous parents. We asked the participants to declare having any medical condition and whether they used to take any medication. Age was divided into groups according to the national classification that was approved by Damascus University
- Other definitions: Respiratory disease in this study was indicate to chronic bronchitis and chronic obstructive pulmonary disease (COPD). We defined low educational level as having a high school degree or less.

### 2.3. Ethical Commute Approval and Consents

Informed consent was taken from the participant before proceeding with the survey for participating in the research and for using and publishing the data. Confidentiality was assured and no questions indicative for the person were asked.

Our study protocol and ethical aspect were reviewed and approved by Damascus University deanship, Damascus, Syria.

### 2.4. Data Process

Data was processed using IBM SPSS software version 26 for Windows (SPSS Inc, IL, USA). Chi-square and one-way ANOVA tests were performed to determine statistical significance between the groups. Pearson's correlation was also calculated. We calculated odds ratios (ORs) and the 95% confidence intervals for the groups using the Mantel–Haenszel test by using the same software. Values of less than 0.05 for the two-tailed *P* values were considered statistically significant.

## 3. Results

### 3.1. Characteristics of the Sample

Our study included 968 subjects with 247 (25.5%) being male and 721 (74.5%) being female. The characteristics of the subjects are demonstrated in Table 1. The mean age was 24.69 ± 7.603years (CI 95%: 24.23-25.19). The mean SES score was 14.75 ± 5.280 (14.42-15.09 at CI = 95%). The mean SFAR score was 6.34 ± 3.649 (CI 95%: 6.11-6.57) and the percentage of subjects with AR was 47.9% (CI 95%: 44.7%-51.1%). Characteristics of war, the current medical conditions and medications, and SFAR score in the subjects are demonstrated in Table 2. Among university students, the mean SFAR score was 6.30 ± 3.651 (CI 95%: 5.98-6.59), and the valid percentage of subjects with AR was 47.4% (CI 95%: 43.6%-51.3%). There was no statistically significant difference between university students and the other subjects (*P* > 0.05).

### 3.2. AR Correlations with Other Factors

Comparing subjects with positive or negative AR is demonstrated in Table 3. We found that being male was correlated with having AR [*P* = 0.001 (OR, 1.677; 95% CI 1.249-2.253)]. Having a job was also correlated with having AR more frequently [*P* = 0.049 (OR, 1.309; 95% CI 1.001-1.713)]. However, it was not correlated with any type of work (*P* > 0.05). Having other medical conditions was also correlated with having AR (*P* < 0.0001), especially with having other allergic reactions [*P* < 0.0001 (OR, 9.199; 95% CI 3.836-22.063)] and having asthma [*P* = 0.006 (OR, 5.060; 95% CI 1.396-18.342)]. This was also the case with being distressed from war noises as it was correlated with having AR more frequently [*P* = 0.027 (OR, 1.348; 95% CI 1.034-1.757)].

Furthermore, subjects who had AR took more medications, either prescribed or OTC (*P* < 0.0001). However, we did not find a statistically significant difference when comparing having AR with age, consanguinity, SES, educational level, cigarette and shisha smoking, and losing someone or changing place of living due to war (*P* > 0.05). When Pearson's correlation was calculated, no correlation was found between SFAR scores and age, SES scores, and the number of times of changing place of living due to war. No correlation was found with SES classification and SFAR scores when one-way ANOVA was used (*P* > 0.05).

### 3.3. AR Symptoms

The mean scores and prevalence of each symptom of the SFAR items in positive AR subjects are demonstrated in Table 4. More than 65% of subjects had symptoms of sneezing, blocked nose, runny nose, and itchy eyes. Pollen season allergy was declared by 25.2% and perennial allergy by 48.1%. 75.2% of AR subjects declared that pollen, house dust, or mite triggered their symptoms.

### 3.4. AR Distribution in Provinces

**Figure 1:**
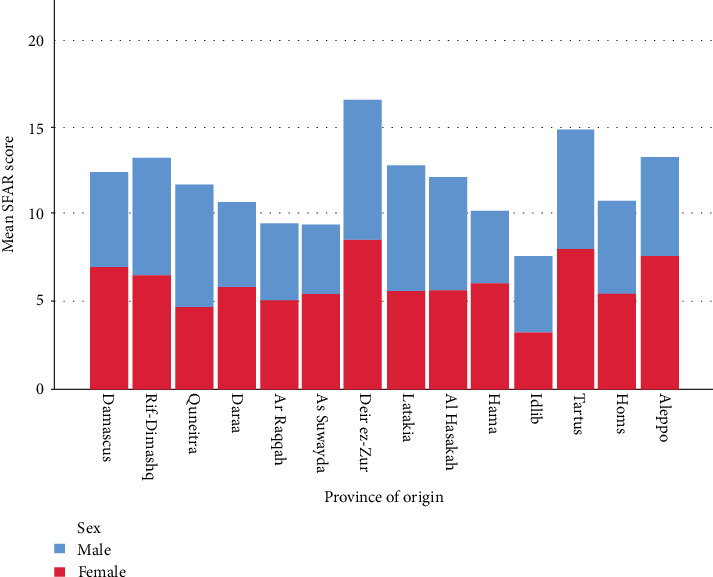
Showing male and female distribution in provinces with their mean SFAR score.

A statistically significant difference was found when comparing having AR with the province of origin (*P* = 0.005) and the province of currently living (*P* = 0.010) with Idlib, Daraa, and As-Suwayda having less AR but Tartus and Deir ez-Zur as provinces of origin having more AR. Furthermore, Hama, Idlib, Homs, Daraa, and As-Suwayda as provinces of current living had less frequency of AR (Figure 1).

## 4. Discussion

### 4.1. AR Categorisation and Burden

AR has been previously categorised as perennial, intermittent, and occupational. However, the new classification is having symptoms either intermittently or persistently [16–18]. We found that AR is associated with being employed, but not to a specific type of work. Although around 75% of subjects declared having allergies from pollen which is in particular seasons in the year, around half of the subjects also declared having allergies throughout the year. AR affects nearly 20 to 40 million people in the United States alone, and these numbers are increasing; an estimated 20% of cases are seasonal, 40% of cases are perennial rhinitis, and 40% of cases are odf both [14]. Risk factors that were highly studied were pollens, drugs, domestic pets, and family history effects on AR [19–21]. The prevalence of AR was found to be approximately 1.4% to 39.7% in 13-14 years old worldwide [22]. In the US, prevalence ranged between 11.9% and 30.2% depending on symptoms and physician diagnosis [23, 24]. In developing countries, AR is especially poorly documented due to a lack of appropriate diagnostic tools [25]. In Europe, AR remains a significant health problem in the community due to the high burden of symptoms and its negative impact on the quality of life, affecting one in five Europeans [26, 27]. It was also found in another study that AR prevalence reached 10% in the Middle East, while in Egypt it was 11% and in Lebanon 9% [28]. However, the prevalence in Syria was much higher, reaching up to 47.9% according to our research which is significantly different from the previous studies (*P* < 0.0001). The high prevalence of AR in Syria could be due to the war as it exposed the population to various substances. We found a statistically significant correlation between AR and distress from war noises. However, this estimation was based on a screening tool rather than a medical diagnosis due to lack of resources, especially at this time of conflict.

### 4.2. AR and Its Correlations

In a regional country close to Syria, there was no statistical difference between having AR and gender, smoking, place of living, and other housing and economic conditions [29]. This was also found in another country in the region where gender, smoking, and domestic exposure did not have a significant correlation with AR, but age and area of residency were found to be correlated [30]. In Turkey, AR was found to be the most common allergic disease, and the prevalence rate of clinical symptoms was 11.4% with a higher rate among females and in urban areas [31]. However, it was found in a review that AR was more closely correlated with male gender, tobacco smoke, aspirin, and higher socioeconomic status [32]. Although smoking was found in Syria to be more common in males [33], AR, which was found to be more common in females, was correlated with smoking. Furthermore, AR was found to be correlated with laryngopharyngeal reflux (LPR) disease [34], the prevalence of LPR was also found to be in more than one-third in on study Syria [35] which could explain the high prevalence of AR.

Another study found that the coexistence of asthma and AR was more common in female adolescents [36]. Another study found a slight female predominance in adulthood with AR [37]. In addition, the risk for AR was inversely correlated with urbanisation [38]. However, many other studies found that the higher the SES, the higher AR symptoms were [39–41], but asthma correlation with SES had conflicting data [41, 42]. Smoking was not found to alter nasal symptoms of AR or quality of life [43] although a significant increase of self-reported rhinitis symptoms was found in adult smokers [44]. In our study, AR had a female predominance. However, with the previous conflicting data, our results showed that smoking shisha or cigarettes, SES, and educational level were not correlated with AR although we cannot to compare Syrian SES with the afore mentioned studies as there is a significant gap between SES levels in Syria and the population of the previouse studies.

In contrast, being employed increased the prevalence of AR in our subjects. However, no significant association was found with any particular type of employment. We also found a significant difference in AR prevalence depending on the province of residence. AR was found to increase the risk of asthma and atopic diseases.

### 4.3. The Significance of the Findings

When AR is not aggressively and early treated, it can lead to an increase in the incidence of asthma [32]. This was also found in our study as subjects with AR reported having asthma and allergies more often. Such as high prevalence can have severe ramifications in the future and can add to suffer from people and make the medical sector suffers even further.

While rates of diagnosis and treatment appear to be high worldwide, there are few reports on patient satisfaction and potential unmet needs in AR sufferers. There is also a need for more data on the diagnosis of respiratory allergies, the use of symptomatic medications, and the potential role of a guideline-recommended treatment option, for example, allergen immunotherapy (AIT) [28, 45, 46]. Distress from war noise was found to be correlated with AR and was found in another study to be correlated with mental distress and that over than 75% of participants were distressed from war noise [47]. We need to dedicate many resources to the health sector in Syria and upgrade the current system, so we can maximise the efficacy and enable better care, mainly as Syria has unique environment and population practices and habits may expose them to harmful substances that may be the cause of the high prevalence of allergies and AR [48].

## 5. In Conclusion

AR has complicated interactions with different risk factors and increases the risk of asthma if left untreated. It can also severely affect the quality of life. AR has a particularly high prevalence in the Syrian population which suggests underlying factors leading to this incline. This evidence reinforces the importance of studying AR and its risk factors, particularly in Syria, in order to have a comprehensive approach to treatment for such a prevalent health issue which contributes a significant burden on the health sector.

## 6. Limitations

- This study was online based and, therefore, could not exactly determine the studied population. AR diagnosis was based on a screening tool, not a medical diagnosis. However, the effect of these two issues was minimized by the sample size and the high prevalence of AR found as it is a reflective result of the problem
- Smoking could only be simply addressed and correlated with AR
- No further details could be collected on SES and war-related events as this may raise some concerns and it would not be accepted by Damascus University ethical commutee as it will not be nationally acceptable questions. SES measurement is also different from other countries as the average income and standards are different,

## Figures and Tables

**Table 1:** Characteristics of subjects and their demographic data.

Characteristic	Frequency (*n* = 968)	Percentage%
Age		
0–17	21	2.2
18–30	825	85.2
31–45	96	9.9
46+	26	2.7
Gender		
Male	247	25.5
Female	721	74.5
Place of living		
Damascus	525	54.2
Rif-Dimashq	50	5.2
Aleppo	62	6.4
Homs and Hama	127	14.3
Al-Jazira region	5	0.6
Southern Syria	24	2.7
Syrian coast	94	10.6
Idlib	4	0.4
Smoking cigarettes		
No	822	84.9
Yes regularly	146	15.1
Smoking shisha		
No	686	71.0
Yes regularly	280	29.0
Educational level		
Primary school	1	0.1
High school	61	6.3
Intermediate or higher Institute certificate	767	79.5
Master or PhD	136	14.1
SES level		
Intermediate or higher Lower	24	2.5
Upper lower	215	22.2
Lower middle	242	25.0
Upper middle	466	48.1
Upper	21	2.2
Employment status		
Unemployed	615	63.9
Employed	348	36.1

**Table 2:** Other characteristics of war, the current medical conditions and medications, and SFAR score in the subjects.

Characteristic	Count	Percentage%
Changing the living area		
No	474	49.6
Yes, but not due to the war	178	18.6
Yes	304	31.8
A relative being endangered by the war		
No	307	32.1
Yes	650	67.9
Losing someone due to the war		
No	550	57.4
Yes	408	42.6
Being afraid of the war sounds		
No	347	36.1
Yes	614	63.9
Medical condition		
No	546	64.7
Digestive	76	9.0
Pulmonary	7	0.8
Cardiac	16	1.9
Endocrine	71	8.4
Urinary	12	1.4
Neurological	26	3.1
Skeletal	30	3.6
Asthma	14	1.7
Allergic reaction	46	5.5
Drugs		
No	510	59.1
Yes, some the supplements	45	5.2
Yes, over the counter drugs	83	9.6
Yes, prescribed drugs	225	26.1
AR		
No	504	52.1
Yes	464	47.9

**Table 3:** Comparing subjects with positive and negative AR with other factors.

Characteristic	Positive SFAR score	Percentage (CI 95%)	Negative SFAR score	Percentage (CI 95%)	*P* value
Gender					
Male	95	9.8	152	15.7	0.001
Female	369	38.1	352	36.4	
Consanguinity					
Negative	349	75.9	380	76.3	0.422
Positive	111	24.1	118	23.7	
SES					
Lower	12	2.6	12	2.4	0.880
Upper lower	100	21.6	115	22.8	
Lower middle	113	24.4	129	25.6	
Upper middle	227	48.9	239	47.4	
Upper	12	2.6	9	1.8	
Educational level					
Low	28	6.0	34	6.8	0.646
High	435	94.0	468	93.2	
Cigarette smoking					
No	395	85.1	427	84.7	0.860
Yes daily	69	14.9	77	15.3	
Shisha smoking					
No	319	69.0	367	72.8	0.197
Yes regularly	143	31.0	137	27.2	
Age groups					
0–17	8	1.7	13	2.6	0.886
18–30	396	85.3	429	85.1	
31–45	47	10.1	49	9.7	
46+	13	2.8	13	2.6	
Employment status					
Unemployed	283	62.1	332	68.2	0.049
Employed	173	37.9	155	31.8	
Type of work					
Labourer	11	6.4	16	10.4	0.289
Clerk or in a restaurant	14	8.2	15	9.7	
Technician	58	33.9	45	29.2	
Specialist	82	48.0	71	46.1	
Employee	6	3.5	7	4.5	
Medical condition					
Negative	230	56.7	316	72.1	<0.0001
Digestive	42	10.3	34	7.8	
Respiratory	3	0.7	4	0.9	
Cardiac	8	2.0	8	1.8	
Endocrine	38	9.4	33	7.5	
Urinary	6	1.5	6	1.4	
Neurological	14	3.4	12	2.7	
Skeletal	14	3.4	16	3.7	
Asthma	11	2.7	3	0.7	
Allergic reaction	40	9.9	6	1.4	
Drugs taken					
No	210	51.6	300	65.8	<0.0001
Over the counter	61	15.0	67	14.7	
Prescribed	136	33.4	89	19.5	
Losing someone close due to war					
No	270	61.2	280	58.9	0.758
Yes a loved one or loved friend	4	0.9	2	0.4	
Yes a relative	167	37.9	193	40.6	
Distress from war noises					
Negative	150	32.5	197	39.4	0.027
Positive	311	67.5	303	60.6	
Changing place of living due to war					
Negative	322	70.2	330	66.4	0.213
Positive	137	29.8	167	33.6	

CI: confidence interval.

**Table 4:** Mean score for each SFAR item in subjects with AR.

Characteristic	Mean ± SD	CI (95%)
Sneezing	0.64 ± 0.479	0.465–0.489
Runny nose	0.65 ± 0.478	0.462–0.489
Blocked nose	0.66 ± 0.474	0.457–0.487
Nasal symptoms plus itchy eyes	1.41 ± 0.911	0.867–0.946
Time of occurrence	1.21 ± 0.839	0.808–0.865
Triggers	1.85 ± 0.783	0.718–0.842
Perceived allergic status	0.81 ± 0.396	0.369–0.423
Result of the allergic test	0.65 ± 0.481	0.427–0.503
Previous medical diagnosis	0.62 ± 0.487	0.474–0.495
Familial history of allergy	1.53 ± 0.846	0.791–0.895

## Data Availability

Data can be made available upon request.

## References

[1] Bousquet J., Khaltaev N., Cruz A. A. (2008). Allergic rhinitis and its impact on asthma (ARIA) 2008 update (in collaboration with the World Health Organization, GA(2)LEN and AllerGen). *Allergy*.

[2] Annesi-Maesano I., Didier A., Klossek M., Chanal I., Moreau D., Bousquet J. (2002). The score for allergic rhinitis (SFAR): a simple and valid assessment method in population studies. *Allergy*.

[3] Woolcock A. J., Bastiampillai S. A., Marks G. B., Keena V. A. (2001). The burden of asthma in Australia. *The Medical Journal of Australia*.

[4] Beasley R. (1998). Worldwide variation in prevalence of symptoms of asthma, allergic rhinoconjunctivitis, and atopic eczema: ISAAC. *The Lancet*.

[5] Trikojat K., Buske-Kirschbaum A., Plessow F., Schmitt J., Fischer R. (2017). Memory and multitasking performance during acute allergic inflammation in seasonal allergic rhinitis. *Clinical & Experimental Allergy*.

[6] Li C. W., Chen D. H., Zhong J. T. (2014). Epidemiological characterization and risk factors of allergic rhinitis in the general population in Guangzhou City in China. *PLoS One*.

[7] Lim F. L., Hashim Z., Than L. T. L., Md Said S., Hisham Hashim J., Norbäck D. (2015). Asthma, airway symptoms and rhinitis in office workers in Malaysia: associations with house dust mite (HDM) allergy, cat allergy and levels of house dust mite allergens in office dust. *PLoS One*.

[8] Phathammavong O., Ali M., Phengsavanh A. (2008). Prevalence and potential risk factors of rhinitis and atopic eczema among schoolchildren in Vientiane capital, Lao PDR: ISAAC questionnaire. *BioScience Trends*.

[9] Ziyab A. H. (2017). Prevalence and risk factors of asthma, rhinitis, and eczema and their multimorbidity among young adults in Kuwait: a cross-sectional study. *BioMed Research International*.

[10] Donohue K. M., Miller R. L., Perzanowski M. S. (2013). Prenatal and postnatal bisphenol A exposure and asthma development among inner-city children. *Journal of Allergy and Clinical Immunology*.

[11] Goronfolah L. (2016). Aeroallergens, atopy and allergic rhinitis in the Middle East. *European Annals of Allergy and Clinical Immunology*.

[12] Waibel K. H. (2005). Allergic rhinitis in the Middle East. *Military Medicine*.

[13] John L. J., Ahmed S., Anjum F. (2014). Prevalence of allergies among university students: a study from Ajman, United Arab Emirates. *ISRN Allergy*.

[14] Skoner D. P. (2001). Allergic rhinitis: definition, epidemiology, pathophysiology, detection, and diagnosis. *The Journal of Allergy and Clinical Immunology*.

[15] Alharethy S., al Wedami M., Syouri F. (2017). Validation of the Arabic version of the score for allergic rhinitis tool. *Annals of Saudi Medicine*.

[16] Bousquet J., Khaltaev N., Cruz A. A. (2008). Allergic rhinitis and its impact on asthma (ARIA) 2008. *Allergy*.

[17] Bousquet J., van Cauwenberge P., Khaltaev N. (2001). Allergic rhinitis and its impact on asthma. *Journal of Allergy and Clinical Immunology*.

[18] Cingi C., Songu M., Ural A. (2011). The score for allergic rhinitis study in Turkey. *American Journal of Rhinology & Allergy*.

[19] Kuyucu S., Saraclar Y., Tuncer A. (2006). Epidemiologic characteristics of rhinitis in Turkish children: the International Study of Asthma and Allergies in Childhood (ISAAC) phase 2. *Pediatric Allergy and Immunology*.

[20] Sultész M., Katona G., Hirschberg A., Gálffy G. (2010). Prevalence and risk factors for allergic rhinitis in primary schoolchildren in Budapest. *International Journal of Pediatric Otorhinolaryngology*.

[21] Tamay Z., Akcay A., Ones U., Guler N., Kilic G., Zencir M. (2007). Prevalence and risk factors for allergic rhinitis in primary school children. *International Journal of Pediatric Otorhinolaryngology*.

[22] Strachan D., Sibbald B., Weiland S. (1997). Worldwide variations in prevalence of symptoms of allergic rhinoconjunctivitis in children: the International Study of Asthma and Allergies in Childhood (ISAAC). *Pediatric Allergy and Immunology*.

[23] Nathan R., Meltzer E., Seiner J., Storms W. (1997). Prevalence of allergic rhinitis in the United States. *Journal of Allergy and Clinical Immunology*.

[24] Nathan R. A., Meltzer E. O., Derebery J. (2008). The prevalence of nasal symptoms attributed to allergies in the United States: findings from the burden of rhinitis in an America survey. *Allergy and Asthma Proceedings*.

[25] Piau J. P., Massot C., Moreau D. (2010). Assessing allergic rhinitis in developing countries. *The International Journal of Tuberculosis and Lung Disease*.

[26] Canonica G. W., Bousquet J., Mullol J., Scadding G. K., Virchow J. C. (2007). A survey of the burden of allergic rhinitis in Europe. *Allergy*.

[27] Maurer M., Zuberbier T. (2007). Undertreatment of rhinitis symptoms in Europe: findings from a cross-sectional questionnaire survey. *Allergy*.

[28] Abdulrahman H., Hadi U., Tarraf H. (2012). Nasal Allergies in the Middle Eastern Population: Results from the “Allergies in Middle East Survey”. *American Journal of Rhinology & Allergy*.

[29] Musmar S. (2009). Prevalence of Allergic Rhinitis & Its Risk Factors Among An-Najah University Students - Nablus/Palestine. *Middle East Journal of Family Medicine*.

[30] Salarnia S., Momen T., Jari M. (2018). Prevalence and Risk Factors of Allergic Rhinitis in Primary School Students of Isfahan, Iran. *Advanced Biomedical Research*.

[31] Başak O., Başak S., Gültekin B., Tekin N., Söylemez A. (2006). The prevalence of allergic rhinitis in adults in Aydin, Turkey. *Rhinology*.

[32] Varshney J., Varshney H. (2015). Allergic rhinitis: an overview. *Indian Journal of Otolaryngology and Head & Neck Surgery*.

[33] Kakaje A., Alhalabi M. M., Alyousbashi A., Ghareeb A., Hamid L. (2020). *Smoking Habits in Syria and the Influence of War on Cigarette and Shisha Smoking*.

[34] Kakaje A., Alhalabi M. M., Alyousbashi A., Hamid A., Aldeen O. H. (2020). *Allergic Rhinitis, Asthma and Gastro-Esophageal Reflux Disease: A Cross-Sectional Study on their Reciprocal Relations*.

[35] Kakaje A., Alhalabi M. M., Alyousbashi A., Hamid A., Mahmoud Y. (2020). *Laryngopharyngeal reflux disease in war-torn syria and its association with smoking and other risks*.

[36] Fröhlich M., Pinart M., Keller T. (2017). Is there a sex-shift in prevalence of allergic rhinitis and comorbid asthma from childhood to adulthood? A meta-analysis. *Clinical and Translational Allergy*.

[37] Pinart M., Keller T., Reich A. Sex differences in the prevalence of rhinitis: A systematic review and meta-analysis.

[38] Christensen S. H., Timm S., Janson C. (2016). A clear urban–rural gradient of allergic rhinitis in a population-based study in Northern Europe. *European Clinical Respiratory Journal*.

[39] Mercer M. J., Joubert G., Ehrlich R. I. (2004). Socioeconomic status and prevalence of allergic rhinitis and atopic eczema symptoms in young adolescents. *Pediatric Allergy and Immunology*.

[40] Torfi Y., Bitarafan N., Rajabi M. (2015). Impact of socioeconomic and environmental factors on atopic eczema and allergic rhinitis: a cross sectional study. *EXCLI Journal*.

[41] Uphoff E., Cabieses B., Pinart M., Valdés M., Antó J. M., Wright J. (2015). A systematic review of socioeconomic position in relation to asthma and allergic diseases. *European Respiratory Journal*.

[42] Bråbäck L., Hjern A., Rasmussen F. (2005). Social class in asthma and allergic rhinitis: a national cohort study over three decades. *European Respiratory Journal*.

[43] Bousquet P. J., Cropet C., Klossek J. M., Allaf B., Neukirch F., Bousquet J. (2009). Effect of smoking on symptoms of allergic rhinitis. *Annals of Allergy, Asthma & Immunology*.

[44] Shargorodsky J., Garcia-Esquinas E., Galán I., Navas-Acien A., Lin S. Y. (2015). Allergic Sensitization, Rhinitis and Tobacco Smoke Exposure in US Adults. *PLoS One*.

[45] Canonica G. W., Bousquet J., Casale T. (2009). Sub-lingual immunotherapy: world allergy organization position paper 2009. *Allergy*.

[46] Canonica G. W., Cox L., Pawankar R. (2014). Sublingual immunotherapy: World Allergy Organization position paper 2013 update. *World Allergy Organization Journal*.

[47] Kakaje A., Zohbi R. A., Aldeen O. H., Makki L., Alyousbashi A., Alhaffar B. A. (2020). *Mental disorder and PTSD in Syria during wartime: a national-wide crisis*.

[48] Kakaje A., Alhalabi M. M., Ghareeb A., Karam B., Hamid A., Mansour B. (2020). *Breastfeeding and acute lymphoblastic leukaemia: potential leukemogenesis in children in developing countries*.

